# NEWBORN HEARING SCREENING

**DOI:** 10.1590/S1808-86942010000100001

**Published:** 2015-10-17

**Keywords:** hearing loss, hearing tests, newborn hearing screening

Dear colleague,

In recent years we have seen a special attention being given to disabled individuals in all areas of society and public health care. Early diagnosis, treatment, rehabilitation and prevention policies have involved legislators, managers and multidisciplinary health care professionals with the aim of reducing as much as possible the, often times, devastative effects disabilities can cause.

Hearing development follows gradual stages of complexity, already starting in the uterus. Thus, for a child to acquire language and develop speech, this child must be able to detect sounds, locate them, discriminate them, memorize them, recognize them and, finally, understand them. Any of these stages, especially the early ones, are very important for the process to complete. Its interruption can cause important functional loss to the child's development. Thus, action must be taken as soon as possible in order to minimize the difficulties brought about by sensorial disorders.

In developed countries, sensorineural hearing loss affects 1 in every 1,000 newborns, and 40% of the cases may be due to hereditary factors, 30% to the many acquired etiologies and 20% still have unknown etiology. The Central Nervous System has great plasticity, when stimulated earlier on, especially before 12 months of age, enabling an increase in nervous connections and providing better results in the auditory rehabilitation and language development of children affected by hearing impairment. The first six months of life are decisive for the child's future development, and for these reasons otorhinolaryngologists, speech and hearing therapists and pediatricians have been concerned with campaigns to bring awareness to the population and health care professionals about the importance of an early identification and diagnosis of hearing loss, immediately following the medical and speech and hearing intervention measures.

Because of the increase in the number of hospitals implementing Universal Neonatal Hearing Screening Programs (UNHS), and also because of the approval of municipal and state laws, we decided to give a professional opinion on UNHS, aiming at guiding the actions of the health care professionals involved.

The multiprofessional Committee on Hearing Health (COMUSA), created in 2007, is a committee that involves Speech and Hearing Therapists, Otologists, Otorhinolaryngologists and Pediatricians. Its goals are to discuss and recommend actions regarding the hearing health of newborns, infants, pre-school aged children, school-aged children, teenagers, adults and the elderly. During two years we discussed, studied and created a text initially considering the UNHS. COMUSA is made up of representatives from the Brazilian Academy of Audiology (ABA), Brazilian Association of Otorhinolaryngology and Neck and Facial Surgery (ABORL), Brazilian Society of Speech and Hearing Therapy (SBFa), Brazilian Society of Otology (SBO) and Brazilian Society of Pediatrics (SBP), all signing this document we present in this issue of our Journal.

I hope reading this text can help minimize and prevent the effects of hearing impairment in children and involve the largest possible number of otorhinolaryngologists in these programs.

Enjoy!

*Prof. Dr. Silvio Antonio Monteiro Marone Full Professor of Otorhinolaryngology – Medical School – Catholic University of Campinas (S. P.)*

*PhD in Otorhinolaryngology – University of São Paulo Medical School.*

